# Illegitimate

**Published:** 1914-09

**Authors:** 


### Illegitimate.

The following editorial from the July number of the *Medical Review of Reviews* is very significant of the radical change in public opinion regarding questions of marriage and the rights of the child.

The author says: "In the general plan for the welfare of mothers and children, one phase of the problem has received inadequate attention. The problem of the child born out of wedlock, destined by an unsympathetic public in legal phraseology to be stigmatized throughout his life, warrants reconsideration.

"The unmarried mothers—those appealing figures claiming the pity of an unthinking community—are surrounded with a veritable martyrdom which they do not deserve. The grace of maternity has been theirs, accompanied with the disgrace of not having the legal sanction far maternity.

"The Boston Children's Aid Society has recognized the fact that the unmarried mother represents a type of human being whose interests have been cast aside thoughtlessly, indifferently, from piety or impiety, from a supercilious arrogance or an ignorant moral hypersensitiveness. It has set an excellent example for other agencies by endeavoring to create a more general understanding of the social significance of illegitimacy.

"From a legal standpoint, illegitimacy appears to be temporarily fixed as a crime. The status of unmarried mothers in the light of the law manifests little justice. These women are creatures with limitations and frailties, but still human and integral portions of the very community which has permitted them to become or made them what they are.

"Granted that many are mentally defective or morally delinquent, they do not deserve to have the burden cast upon them with deadening weight. If mentally defective, they deserve the protection of the public. If institutional care is necessary for the protection of society from the birth of possibly defective children, the responsibility for permitting such mentally sick persons to be exposed to sexual dangers rests upon the community.

"If a lack of education plays a part in their creation, the public is responsible. If economic stress forces some to depart from the line of conduct recognized as legal, the public is responsible. If, friendless, disheartened, untrained and diseased, they are permitted to struggle against all the moral forces of the community, bearing the burden of shame marked by fingers of scorn, but still seeking to care for their offspring, the responsibility for their deadly existence belong to the public. If the lash cut into their flesh, while the fathers of their children escape all responsibility for the support of their offspring, the public is responsible. If the unmarried mothers failed to receive proper obstetrical attention and convalescent care, and their children are not safeguarded in health; if they are given no opportunity to be socially rehabituated, so as to join the ranks of useful workers in the world, society is responsible.

"If the causes of illegitimacy are to be found in inadequate education, low standards of living, a lack of recreation facilities, over-work and under-pay and a limited knowledge of sex hygiene; if, in brief, the causes of illegitimacy are bound up in the pathological functioning of society, why should the product be castigated, abused, condemned, and cast out? Here, indeed, is a medical social problem which deserves the most thoughtful consideration by ministers, physicians, lawyers and legislators. Immorality is not the basis of these shattered lives, wrecked constitutions, and diseased bodies. Perchance, mental defects play a considerable part in lessening the power of sexual resistance on the part of these unfortunates. A lack of education and training also constitutes a marked factor in securing the immoral state of mind which is productive of such disastrous results. These, however, are not faults to be blamed upon the victims. If the unmarried mother represents a symptom of the shortcomings in our social system, then the responsibility of the public is all the more definite.

"The community indeed bears a tremendous burden in the wasted lives of mothers and in the death or support of the rejected children. Our boasted charity has been uncharitable. Our education has not fulfilled its traditions. Our medical and psychological knowledge has not been properly applied. Our legislation has not been just. Tinctured with emotionalism and a holier-than-thou attitude, the church and the State have branded the unfortunate victims as unworthy instead of indicating the social responsibility of the community for the existence of unmarried mothers and their hatefully termed illegitimate children."

---

Dr. A. Scuyler Clarke in a paper read before the United Medical Society of New York declared that he had demonstrated to his own satisfaction the effective use of radium in the treatment of skin cancer, if the cases are treated by the single or massive dose. A special point made by the doctor was that when treated by the single massive dose method the cancer healed smoothly and that none of them recur.

---

Value of Mask Over Surgeon's Mouth.—In order to demonstrate the value of a mask over the surgeon's mouth during operations Candler carried out the following experiments with bacillus prodigiosus: With ordinary breathing and quiet speaking no bacilli left the mouth. Coughing for two minutes caused only a few bacilli to be emitted, and these were controlled by a mask of eight layers of gauze, one of four layers being insufficient. Sneezing was shown to be a most dangerous source of infection from the mouth, one sneeze, and that an artificial one, producing 130 colonies of micro-organisms in a circle of 3½ inches diameter at a distance of 18 inches. A mask of eight layers is not sufficient to keep back all organisms during prolonged sneezing, although it reduces the infection considerably. There was, however, no growth on a plate exposed to one sneeze through such a mask.—*Ex.*

